# Necrotic gangrenous intrathoracic appendix in a marfanoid adult patient: a case report

**DOI:** 10.1186/1471-2482-5-4

**Published:** 2005-03-11

**Authors:** Mohannad J Barakat, Jon H Vickers

**Affiliations:** 1 General Surgery, Weston General Hospital, Weston-super-mare, UK

## Abstract

**Background:**

A diaphragmatic hernia is defined as a defect in part of the diaphragm through which abdominal contents can protrude into the thorax. It may be congenital or acquired. In this case report, we aim to demonstrate a congenital diaphragmatic hernia in an adult marfanoid patient which required emergency treatment

**Case presentation:**

A 43 year old woman was admitted with classical appendicitis requiring surgery. She incidentally had Marfan's clinical features with a positive family history for the syndrome. At operation she had grossly abnormal abdominal anatomy. Radiological investigations demonstrated a large right congenital diaphragmatic hernia with an intrathoracic hernial sac containing a perforated gangrenous appendix. The hernial sac was opened surgically and the appendix excised. The patient made a full recovery.

**Conclusion:**

Diaphragmatic hernias are usually congenital in nature often requiring early corrective surgery for future survival. We have demonstrated the presence of an unusually large diaphragmatic defect, almost a hemidiaphragmatic defect, of unknown direct etiology, but of some possible association with Marfan's syndrome in an adult patient presenting with an acute perforated gangrenous appendix requiring emergency life-saving surgery.

## Background

A diaphragmatic hernia is defined as a defect in part of the diaphragm through which abdominal contents can protrude into the thorax. It may be congenital or acquired, usually through trauma [1].

Congenital diaphragmatic hernias usually occur in the posterolateral portion of the diaphragm (Bochdalek's hernia) and are on the left side in 90% of cases. Loops of bowel, even most of the abdominal contents, may protrude into the hemithorax on the involved side [1,5].

Most congenital diaphragmatic hernias are detected in the newborn whereby, after delivery, as the infant cries and swallows air, the loops of bowel quickly fill with air and rapidly enlarge, causing acute respiratory embarrassment as the heart and mediastinal structures are pushed to the right, compressing the right lung. Respiratory distress is immediate in severe cases; a scaphoid abdomen (due to displacement of abdominal viscera into the chest) is likely. Bowel sounds (and an absence of breath sounds) may be heard over the involved hemithorax. In less severe cases, mild respiratory difficulty develops a few hours or days later as abdominal contents progressively herniate through a smaller diaphragmatic defect [1]. Urgent surgery is usually required to repair the defect [1].

Acquired diaphragmatic hernias are relatively rare and result from either blunt or penetrating trauma. Blunt trauma typically produces large radial tears measuring 5–15 cm, most often at the posterolateral aspect of the diaphragm.

Penetrating injuries to the diaphragm can follow accidental trauma, knife or gunshot wounds. Typically, the defect is small, less than two centimeters in size and may present late after years of gradual herniation and enlargement. Occasionally a shotgun blast causes a large defect [3,4].

Diaphragmatic hernias are usually congenital in nature often requiring early corrective surgery for future survival. There have been three recent publications [5-7] in the literature relating Marfan's syndrome to right sided diaphragmatic hernias. Jacobs *et al *[5] described an association between unique FBN1 gene mutations in neonates and the presence of large unilateral diaphragmatic hernias, while Subirats *et al *[7] demonstrated a correlation between patients exhibiting positive Marfan's syndrome features and having unilateral diaphragmatic hernias. Yetman *et al *[5] recently described a case of acute dyspnoea in a child with Marfan's syndrome secondary to bowel herniation into the thoracic cavity. In these published cases, the diaphragmatic hernias were described in neonates or children and always required corrective surgery for survival, unsuccessfully in two cases.

## Case presentation

A 43 year old female district nurse presented to the accident and emergency department with a 6 hour history of initially generalized abdominal pain which was localizing to the right. This was associated with nausea and loss of appetite, made worse by movement and not relieved with intramuscular morphine injection. There was no history of recent or past trauma to the chest or abdomen. On examination, she was tender in the right upper quadrant and right iliac fossa with rebound and guarding, with a Rovsing positive sign and normal bowel sounds. She was incidentally found to have some marfanoid features including the long span of upper limbs, a high arched palate and the very soft early diastolic murmur of aortic regurgitation. The patient had a positive family history of Marfan's syndrome but had never undergone genetic testing to confirm the diagnosis.

**Figure 1:**
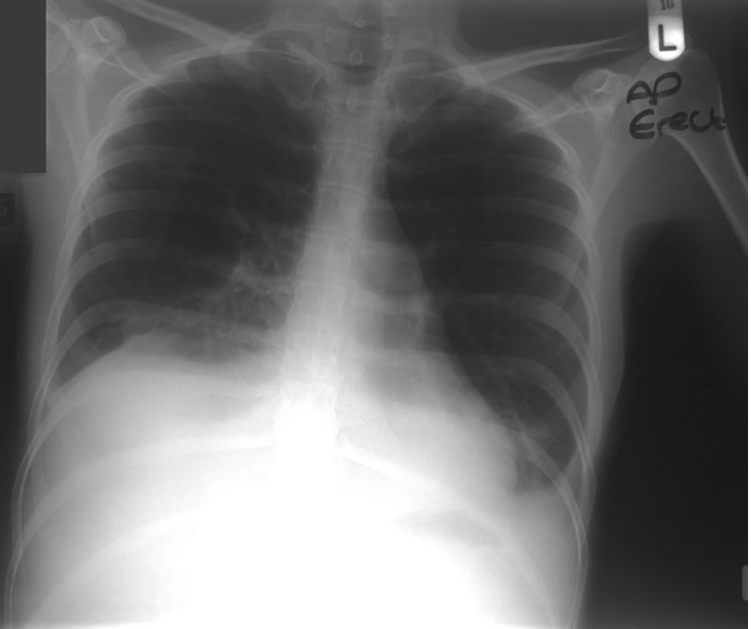
Chest radiograph demonstrating no obvious abnormality

A likely diagnosis of appendicitis was made on the clinical picture associated with a pyrexia and raised white cell count and C – reactive protein. Her abdominal X-ray at that time showed absence of gas in the right side of her bowel. Her chest X-ray did not show any obvious abnormality (Figure 1)

She was taken to theatre the same day for appendicectomy through a standard right iliac fossa incision. There was a small amount of free fluid but the right iliac fossa was filled with a normal looking gall bladder and small bowel only with no sign of the caecum, appendix, ascending or proximal transverse colon. The terminal ileum was found to run up under the right lobe of the liver towards the hilum. The right iliac fossa wound was closed and we proceeded to an exploratory laparoscopy.

At laparoscopy, the liver was retracted to see under the right liver lobe. The proximal transverse colon was found to be running up towards the liver hilum were it felt to be tethered. This suggested a possible herniation of the right colon into the chest. The procedure was abandoned and a CT thorax and abdomen were performed to define the anatomy involved.

**Figure 2:**
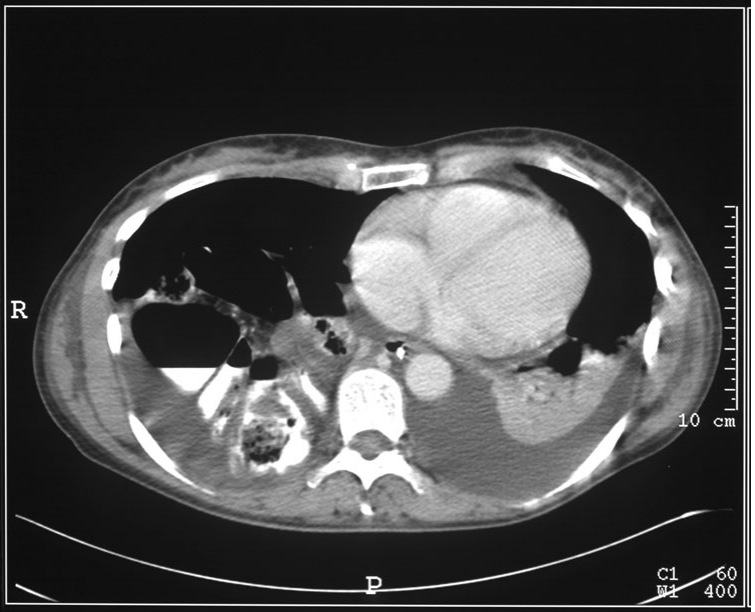
CT scan of thorax and abdomen demonstrating the abnormal anatomy

**Figure 3:**
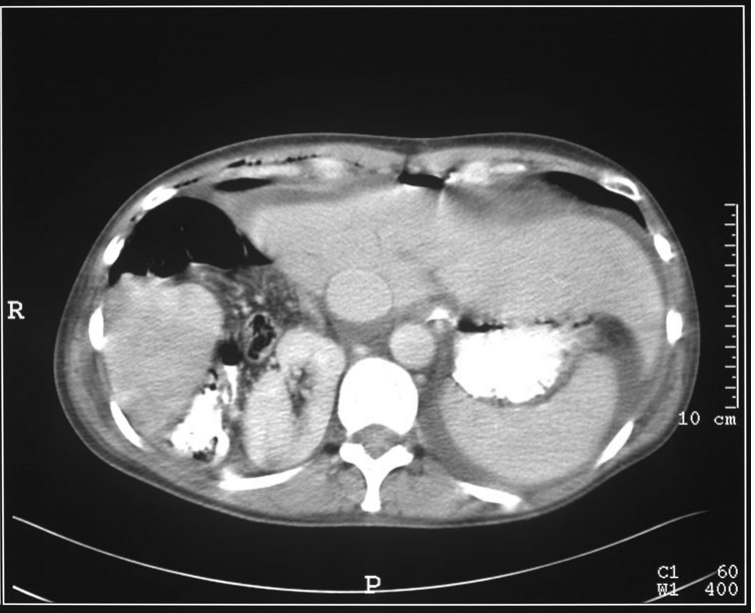
CT scan of thorax and abdomen demonstrating the abnormal anatomy

**Figure 4:**
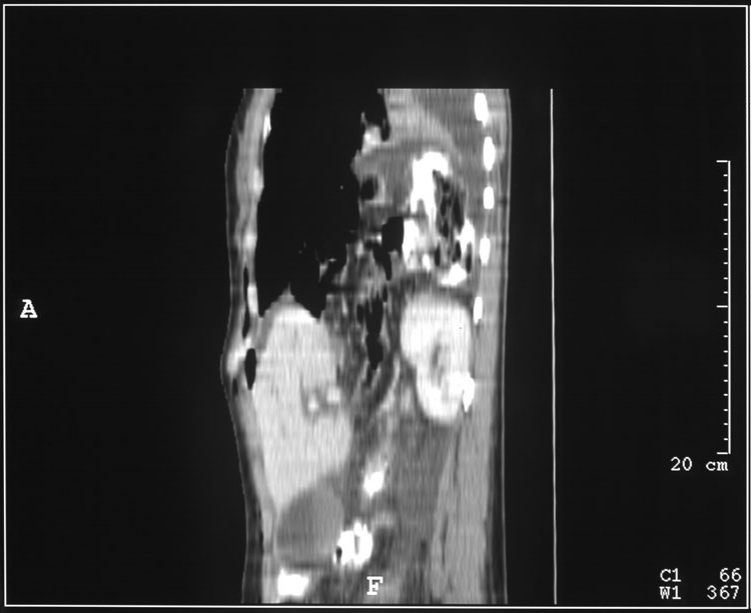
Three-dimensional CT reconstruction demonstrating the diaphragmatic defect

**Figure 5:**
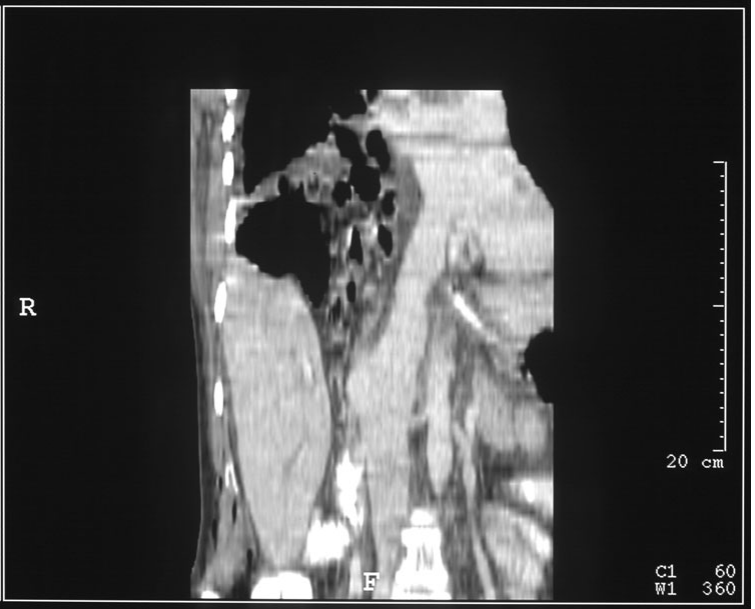
Three-dimensional CT reconstruction demonstrating the diaphragmatic defect

This (Figure 2, 3) demonstrated bilateral pleural effusions of moderate size with underlying unexpanded lungs. The liver had what appeared to be a large Reidl's lobe. Behind the right liver lobe, the right kidney was markedly elevated and the ileo-caecal junction appeared to lie between the liver and the kidney. The right side of the colon appeared to lie above the liver. Three dimensional reconstruction of the scans demonstrated absence of the right hemidiaphragm. (Figure 4, 5)

The symptoms and signs persisted and a laparotomy was performed. At laparotomy, there was some free fluid. There was definitely a large defect in the right hemidiaphragm. The right liver lobe had grown right up into the right intrathoracic space. There was a large hernial sac beneath the right lobe of the liver between the common bile duct, duodenum and liver, displacing the right kidney medially and containing transverse colon, terminal ileum, caecum, appendix and free fluid. The contents of this sac were successfully reduced and a perforated gangrenous appendix with pus was found within the intrathoracic hernial sac. A standard appendicectomy was performed. The right colon was formally mobilized, fully reduced and the caecum was fixed within the right iliac fossa.

**Figure 6:**
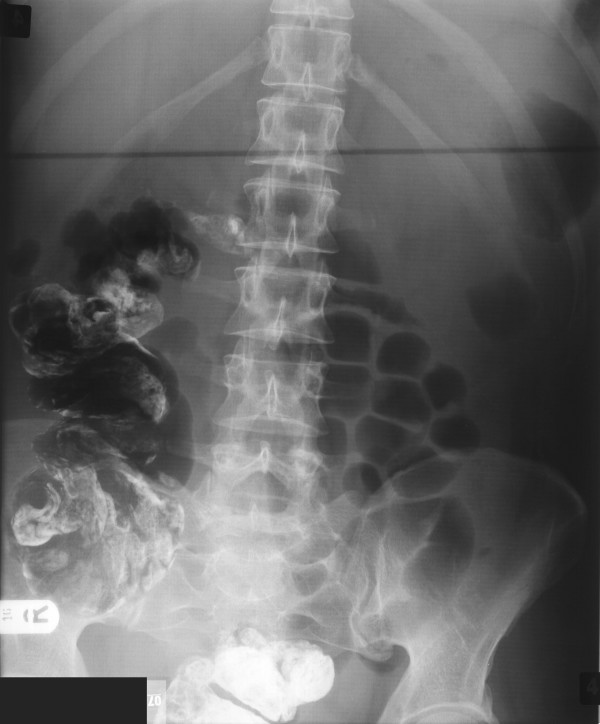
Abdominal radiograph demonstrating the large colon present in the abdominal cavity

The patient was admitted to the intensive care unit postoperatively where she made a good recovery. She had instant relief of her abdominal symptoms and post-operative contrast study demonstrated the presence of the right colon within the abdomen (Figure 6). She was discharged a week post-operatively.

## Conclusion

We have demonstrated a new case of an adult patient, with Marfan's syndrome clinical features and a positive family history of the syndrome, presenting with a large congenital diaphragmatic hernia, compatible with life and undiagnosed into adulthood, requiring an emergency admission into hospital with a perforated gangrenous intrathoracic appendix.

## Abbreviations

CT = computerized tomography

## Competing interests

The author(s) declare that they have no competing interests.

## Authors' contributions

MJB designed the case report, researched the article and drafted the manuscript.

Both MJB and JHV carried out the surgery and were involved in all investigations.

All authors read and approved the final manuscript.

## Pre-publication history

The pre-publication history for this paper can be accessed here:


